# Mobile Health Solutions for Hypertensive Disorders in Pregnancy: Scoping Literature Review

**DOI:** 10.2196/mhealth.9671

**Published:** 2018-05-30

**Authors:** Octavio Rivera-Romero, Alberto Olmo, Rocío Muñoz, Pablo Stiefel, María Luisa Miranda, Luis M Beltrán

**Affiliations:** ^1^ Department of Electronic Technology Universidad de Sevilla Sevilla Spain; ^2^ Instituto de Biomedicina de Sevilla (IBiS) Laboratorio de Hipertensión Arterial e Hipercolesterolemia Servicio Andaluz de Salud / Consejo Superior de Investigaciones Científicas / Universidad de Sevilla Seville Spain; ^3^ Hospital Universitario Virgen del Rocío Sevilla Spain

**Keywords:** pregnancy, hypertension, pre-eclampsia, blood pressure, telemedicine

## Abstract

**Background:**

Hypertensive disorders are the most common complications during pregnancy, occurring in 5% to 11% of pregnancies; gestational hypertension and preeclampsia are the leading causes of perinatal and maternal morbidity and mortality, especially in low- and middle-income countries (LMIC) where maternal and perinatal mortality ratios are still high. Pregnant women with hypertensive disorders could greatly benefit from mobile health (mHealth) solutions as a novel way to identify and control early symptoms, as shown in an increasing number of publications in the field. Such digital health solutions may overcome access limiting factors and the lack of skilled medical professionals and finances commonly presented in resource-poor environments.

**Objective:**

The aim of this study was to conduct a literature review of mHealth solutions used as support in hypertensive disorders during pregnancy, with the objective to identify the most relevant protocols and prototypes that could influence and improve current clinical practice.

**Methods:**

A methodological review following a scoping methodology was conducted. Manuscripts published in research journals reporting technical information of mHealth solutions for hypertensive disorders in pregnancy were included, categorizing articles in different groups: Diagnosis and Monitoring, mHealth Decision Support System, Education, and Health Promotion, and seven research questions were posed to study the manuscripts.

**Results:**

The search in electronic research databases yielded 327 articles. After removing duplicates, 230 articles were selected for screening. Finally, 11 articles met the inclusion criteria, and data were extracted from them. Very positive results in the improvement of maternal health and acceptability of solutions were found, although most of the studies involved a small number of participants, and none were complete clinical studies. Accordingly, none of the reported prototypes were integrated in the different health care systems. Only 4 studies used sensors for physiological measurements, and only 2 used blood pressure sensors despite the importance of this physiological parameter in the control of hypertension. The reported mHealth solutions have great potential to improve clinical practice in areas lacking skilled medical professionals or with a low health care budget, of special relevance in LMIC, although again, no extensive clinical validation has been carried out in these environments.

**Conclusions:**

mHealth solutions hold enormous potential to support hypertensive disorders during pregnancy and improve current clinical practice. Although very positive results have been reported in terms of usability and the improvement of maternal health, rigorous complete clinical trials are still necessary to support integration in health care systems. There is a clear need for simple mHealth solutions specifically developed for resource-poor environments that meet the United Nations Sustainable Development Goal (SDG); of enormous interest in LMIC.

## Introduction

### Background

Hypertensive disorders are the most common complications during pregnancy, occurring in 5% to 11% of pregnancies [1-3]. These incidence rates are showing an increased tendency in some countries [4-9]. There are different types of hypertensive disorders, including gestational hypertension, preeclampsia, chronic hypertension, and preeclampsia superimposed on chronic hypertension [10-12]. Gestational hypertension is the most common cause of hypertension in pregnancy. Preeclampsia is a more severe complication that is often diagnosed after 20 weeks of gestation or within the first 4 to 6 weeks postpartum. It is estimated to affect between 5% and 8% of healthy pregnancies [13]. On the other hand, 10% to 50% of patients with gestational hypertension are diagnosed with preeclampsia 1 to 5 weeks after the diagnosis [14,15].

Gestational hypertension and preeclampsia are the leading causes of perinatal and maternal morbidity and mortality [16-20]. In developed countries, maternal and perinatal mortality ratios are relatively low, but in low- and middle-income countries (LMIC), these ratios are still high. More than 99% of preeclampsia-related maternal deaths occur in LMIC [21]. Nearly one-tenth of all maternal deaths in Asia are associated with hypertensive disorders of pregnancy, whereas one-quarter of maternal deaths in Latin America have been associated with these complications [22]. It is estimated that 9.1% of maternal deaths in Africa are due to hypertensive disorders of pregnancy [23]. Muti et al [24] conducted an analytic cross-sectional study in Zimbabwe and found a pregnancy-induced hypertension prevalence of 19.4%. This ratio was high, and these women were at higher risk of adverse pregnancy outcomes. Tachiwenyika et al [25] also found that pregnancy-induced hypertension was associated with an increased risk of perinatal mortality. Preterm birth complications contribute to a high percentage of under-five mortality causes. As an example, 39% of neonatal deaths are caused by preterm birth complications in Zimbabwe [26]. The Zimbabwe Maternal and Perinatal mortality study of 2007 [27] found pregnancy-induced hypertension to be among the top five causes of maternal mortality and the third highest reason for referral in labor. Placental abruption, disseminated intravascular coagulation, cerebral hemorrhage, and hepatic and renal failure are more likely among pregnant women with gestational hypertension and preeclampsia [11,28]. Preeclampsia is associated with acute kidney function impairments [29,30], a significant number of preterm deliveries, and subsequent neonatal and long-term morbidity [31-33]. Caesarean delivery is more often common among women with a history of preeclampsia than those with an uncomplicated pregnancy history. Bokslag et al [34] found that women with preeclampsia had a shorter gestational age, children with a lower birth weight, and lower placental weight. Moreover, metabolic syndrome is significantly more likely among women with a history of preeclampsia.

Furthermore, gestational hypertension and preeclampsia may also cause long-term effects after pregnancy. Approximately 15% of women with a history of gestational hypertension suffer from chronic hypertension after pregnancy [35]. They are also at increased risk of cardiovascular diseases (CVDs) such as ischemic heart disease and stroke in later life [36-40]. Women experiencing preeclampsia have an increased risk of high blood pressure (BP), cardiovascular complications, kidney disease, diabetes mellitus, thromboembolism, thyroid disease, and impaired memory in later life [36,41-45]. These women have approximately double the risk of cardiovascular events in the 5 to 15 years after pregnancy compared with women who are normotensive during pregnancy [36]. Several studies have demonstrated a greater risk of death from CVD among women who had preeclampsia during pregnancy than those who remained normotensive [36,40,46]. That risk is even greater if the pregnancy ended prematurely [46]. Gestational hypertension and preeclampsia could also affect the offspring’s adult health [47]. In addition, children born to women who had preeclampsia during their pregnancy have an increased risk of CVD [42].

The early recognition of signs and symptoms of such hypertensive disorders may prevent some complications [17,48]. In this sense, pregnant women with hypertensive disorders could benefit from mobile health (mHealth) solutions. Information and communication technology (ICT) could be used to support diagnosis and monitoring, management and self-care, communication between patients and maternity care providers, and patient education and empowerment. Many factors such as genetic predisposition, having a previous history of preeclampsia, obesity, excessive weight gain, elevated BP, multifetal gestation, in vitro fecundation, maternal age, and smoking have been associated with gestational hypertension and preeclampsia [49-55], and a number of mHealth solutions are being reported to better control these parameters.

This control of early symptoms is especially interesting in LMIC settings, with delayed identification of women with hypertensive disorders because of their limited health care capability and facilities for testing and managing such patients [56]. In Zanzibar, a mobile phone intervention reduced perinatal mortality [57], as reported in a cluster randomized controlled trial (RCT) with primary health care facilities. Other recent works in LMIC are targeting hypertensive-related problems from a more initial stage [56]. Digital health may overcome access-limiting factors and the lack of skilled medical professionals and finances. Using ICT-based interventions, a large number of patients could be treated at any time and at reduced health care costs, while the quality and efficiency of care are maintained or even increased. Therefore, there is a clear need for simple mHealth solutions specifically developed for resource-poor environments that meet the United Nations Sustainable Development Goals (SDGs) [58], particularly SDG 3: “Ensure healthy lives and promoting well-being for all at all ages, which among its targets are to reduce the maternal and neonatal mortality ratios.”

### Objectives

A scoping review of mHealth solutions used as a support in hypertensive disorders during pregnancy has been conducted. The objective of this review was to analyze the current state of knowledge of digital health for hypertensive disorders in pregnancy.

## Methods

### Research Questions

A methodological review following a scoping methodology as proposed in the studies by Arksey et al and Levac et al [59,60] has been conducted. This literature review aimed to analyze the state-of-the-art and identify research gaps related to how mobile technology could support hypertensive disorders in pregnancy. Table 1 shows the research questions and the corresponding rationale for each one.

### Search Strategy

An electronic search of the literature was performed in English across the following databases: MEDLINE, PubMed, CINAHL, Science Direct, and the Cochrane Central Registry of Controlled Trials. A combination of keywords involving medical and ICT-related terms was used. The search string was set through an iterative process to include all relevant keywords identified in previous search results. The final search string is shown in Table 2. Bibliographies and reference lists were also scrutinized to identify any other relevant studies. The literature search was performed on March 3, 2017.

### Inclusion Criteria

Textbox 1 shows the inclusion criteria for studies.

### Exclusion Criteria

Textbox 2 shows the exclusion criteria for studies.

### Study Selection

A literature search was performed by the primary author (OR) according to the search strategy described above. Retrieved titles and abstracts were screened to eliminate duplicates. Then, two authors (OR and AO) reviewed the titles and abstracts and filtered out applying inclusion and exclusion criteria. Discrepancies were solved by consensus. Cohen kappa coefficient was calculated to measure interrater agreement (k=0.84) [61]. Full-text articles in the filtered list were obtained, and two authors (OR and AO) reviewed them, excluding those that did not meet the inclusion criteria or the exclusion criteria. When papers reported data regarding the same digital health solution, only one was included in the final analysis. The included papers were assessed for data extraction.

**Table 1:** Research questions.

Research question (RQ)	Rationale
RQ 1: Which mobile health (mHealth) solutions have been used in digital interventions involving patients with hypertensive disorders in pregnancy?	To identify different types of mHealth solutions supporting hypertensive disorders in pregnancy.
RQ 2: What are the areas of focus of these mHealth solutions?	To understand how mHealth solutions supported hypertensive disorders in pregnancy (diagnosis, monitoring, decision support, health promotion, or education).
RQ 3: What were the target groups of these mHealth solutions?	To identify potential users of mHealth solutions supporting hypertensive disorders in pregnancy.
RQ 4: What types of interventions or studies were performed?	To determine whether research in the area has been validated through empirical studies and evidence found through clinical trials.
RQ 5: What were the main outcomes?	To discover the benefits and positive outcomes of mHealth solutions supporting hypertensive disorders in pregnancy.
RQ 6: How is the research focused on mHealth solutions to support hypertensive disorders in pregnancy distributed across countries?	To explore the geographic publication trends of information and communication technology (ICT) health to support hypertensive disorders in pregnancy.
RQ 7: How is the research focused on mHealth solutions to support hypertensive disorders in pregnancy distributed over the last 10 years?	To determine how the trends of mHealth solutions to support hypertensive disorders in pregnancy are evolving.

**Table 2:** Search string.

Scope	String
Health	(Preeclampsia OR eclampsia OR gestational hypertension) AND
Technology	(smartphone OR mobile health OR ehealth OR website OR Internet OR (social AND (net OR media))

**Inclusion criteria.**

- Papers published in research journals reporting technical information of a mobile health (mHealth) solution for hypertensive disorders in pregnancy
- Studies reporting the results from an intervention using a digital health solution for the same domain
- Only papers in English or Spanish
- Publications from the period of January 1, 2017 to March 3, 2017
- Papers reporting data regarding digital health solutions intended for both patients and health professionals

**Exclusion criteria.**

- Papers published in languages other than Spanish or English
- Studies reporting information on other diseases such as gestational diabetes mellitus and population groups other than pregnant women with hypertensive disorders
- Papers published in research avenues other than research journals

### Data Extraction

Three datasets were defined to be extracted from selected papers. As the first dataset, a categorization of mHealth solutions was identified. Initially, three categories were proposed based on those used in digital health reviews performed in other diseases: health promotion, management, and diagnosis. Then, a second category proposal was defined by the consensus of two authors (OR and AO) to identify the most relevant categories related to hypertensive disorders in pregnancy. The resulting categories were as follows: *Diagnosis and Monitoring, mHealth Connected Decision Support Systems (DSSs), Education, and Health Promotion*. The rest of the authors (RM, PS, MLM, and LB) reviewed the categories defined, and concepts were redefined to clarify them when necessary.

The *Diagnosis and Monitoring* category included all mHealth solutions supporting the diagnosis of hypertensive disorders in pregnancy or monitoring patients’ status, including physical or physiological measurements such as BP and the presence of proteinuria. This category was included because there is a recommendation for pregnant women with hypertensive disorders to automatically monitor their BP at home at least twice daily over several days [62]. In addition, measuring weight gain during pregnancy is an important issue that may promote self-care and improve overall engagement with prenatal care [63]. On the other hand, one of the signs that could be used to diagnose preeclampsia or renal disease is proteinuria [62].

Hypertensive disorders in pregnancy must be detected early and appropriately managed to prevent severe complications and potential effects. Predictive personalized models complemented with real-time indicators of the diseases [64] and rapid medical attention should be provided. *mHealth Connected DSSs* are key tools used to improve the quality of health care by enhancing the quality of services and controlling the cost of health care delivery [65]. DSSs can activate alerts when deviation from recommended care is detected or when a new relevant symptom is recognized. Clinician and patient actions may be triggered by these alerts to ensure compliance to clinical care standards and guidelines. There are clinical models with excellent, clinically validated prediction features for many of the most frequent and high-impact diseases in pregnancy [62]. Moreover, there are several sensor technologies that enable the real-time improvement of the predictive capacities of computer models. These existing clinical models and sensor technologies enable the creation of DSSs focused on raising individual awareness and empowering women in the management of their health. They can also be used in the design and development of DSSs supporting health care professionals (HCPs) in the treatment of pregnant women with hypertensive disorders.

The *Education* category was focused on educational computer-based solutions. This category was added because of its relevance for pregnant women with hypertensive disorders. Patients’ knowledge about hypertensive disease may improve their health outcomes, leading them to seek earlier appropriate care as soon as they recognize signs and symptoms [66,67]. Many of them have a poor understanding of the disease process [68]. They should be provided with culturally sensitive information at an appropriate reading level. Pregnant women with hypertensive disorders often have significant psychological consequences such as maternal anxiety. Individual levels of anxiety may persist unnecessarily because of the lack of knowledge of the normal pregnant state and the potential complications of pregnancy [69]. In addition, this category was related to *Diagnosis and Monitoring* because of the involvement of the patient in the self-measurement and interpretation of physiological signals, which needs to be carried out following a specific procedure to obtain trusted measurements. This training can be carried out through educational mHealth-based programs.

The final category is *Health Promotion.* Pregnant women with hypertensive disorders may benefit from lifestyle interventions such as exercise, changes in dietary habits, and smoking cessation [70]. Healthy lifestyle recommendations have been included for pregnant women at low or high risk of preeclampsia [62]. Abstention from alcohol, smoking cessation, exercises for the maintenance of fitness, calcium supplementation, and stress reduction, among others, are recommended to prevent preeclampsia and its complications. Some researchers have found a significant reduction of BP with daily intake of chocolate, resulting in a reduced risk of gestational hypertension and preeclampsia [71,72]. Although previous researchers have found a positive influence of physical activity (PA) on pregnant women and their infants [73-75], there is a lack of clinically validated research aimed to determine the influence of exercise on the risk of hypertensive disorders. However, exercise is included among recommendations for pregnant women with hypertensive disorders because of its positive influence on complications. Healthy lifestyle promotion through ICT may be a powerful and useful tool for pregnant women with hypertensive disorders. In this sense, we included the *Health Promotion* category to classify all ICT health solutions other than DSSs and educational computer-based programs.

The second dataset consisted of technical data on mHealth solutions reported in the selected studies. Data on the type of ICT solution, platform, users, main features, content validation, and communication between patients and HCPs were extracted. The type of ICT solution identified hardware and software used in the studies, including information about affiliation when available. These data were interesting to estimate the cost of the proposed solutions. The information regarding mobile, Web, or medical equipment platforms used in the proposed solution was also gathered. This information was relevant to estimate which solutions could be used in LMIC settings. Potential users of ICT solutions were identified as patients, HCPs, or both. Relevant information about the functionalities and the features implemented was also extracted. Among others, early diagnosis, continuous monitoring, signs and symptoms recognition, and patients’ information needs are relevant issues in hypertensive disorders in pregnancy. Users’ confidence in the contents included in solutions should be high to effectively empower them. We collected data regarding how authors addressed this confidence in their proposals. In addition, communication between patients and HCPs is an important functionality to be implemented in these solutions. Therefore, we defined a special field to gather information about communication functionality.

Finally, a third dataset was related to interventions reported in the included studies if available. Data regarding the type of interventions, sessions, participants, inclusion criteria, the context in which the interventions were performed, countries in which they were conducted, scales used, measurements collected, and outcomes found were extracted.

Two reviewers (OR and AO) extracted data from the selected papers. Both collected data independently from these selected studies. Differences were discussed, and decisions were made by consensus. The results were then discussed with the medical team (RM, PS, MLM, and LB) to analyze clinical relevance and potential use of mHealth solutions in the health care system.

## Results

### Included Studies

**Figure 1:**
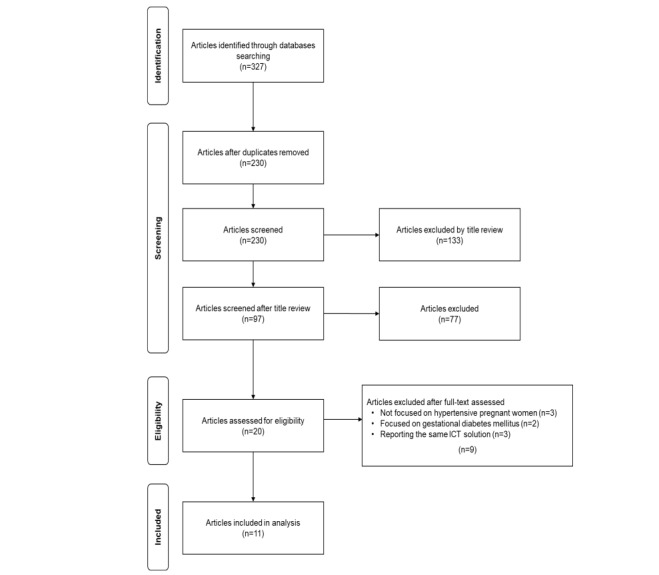
Flow diagram for the inclusion of the studies reporting information and communication (ICT) solutions for hypertensive disorders in pregnancy.

The search in electronic research databases yielded 327 articles. After removing duplicates, 230 articles were selected for screening. Due to the number of articles, a title review excluded 133 articles. Then, the titles and abstracts of 97 selected articles were obtained; 77 of them were excluded because they did not meet the inclusion criteria. Full-text publications were obtained for the resulting 20 articles; 9 were excluded because of the following reasons: different population group (pregnant women without hypertensive disorders or postpartum women) [76-78], other disorders (gestational diabetes mellitus, GDM) [79,80], and the same mHealth solution [81-84]. Finally, data extraction was performed on the final 11 included articles. A Preferred Reporting Item for Systematic Reviews and Meta-Analyses flow diagram representing the full process described above is shown in Figure 1, and detailed information is presented in Multimedia Appendix 1.

### Description of the Included Solutions

Walker et al [69] proposed an educational website to empower women with high-risk pregnancy. In the website, the information related to the placental complications of pregnancy was provided. Researchers defined an education strategy based on this website and studied its impact on the maternal knowledge of the placental complications of pregnancy and on the maternal anxiety of women with high-risk pregnancy. The intervention significantly improved the patient knowledge and significantly reduced the maternal anxiety.

Peleg et al [84] established a ubiquitous, user-friendly, and patient-centered mobile DSS for both patients with chronic conditions and for their care providers. The system was based on clinical guidelines and monitored continuously the patients’ heath state using mobile sensors and self-reporting of symptoms. Patients’ preferences and psychosocial and demographic context was considered by the system to empower the patients and increase their compliance. In addition, a semantic data integration into a personal health record was implemented based on a generic architecture supporting interoperability of different devices and electronic health records. The feasibility of the system and some of the potential benefits of the evidence-based distributed patient-guidance system were tested with a first prototype.

Berlage et al [85] created the GerOSS platform aimed at generating a deeper insight into the relevant risk factors to improve the diagnosis and the treatment of severe complications during pregnancy and delivery. The GerOSS platform contained a database with information that was collected through an Internet-based system. This information included the reporting and the documentation of rare obstetric events. The data were analyzed by experts, and the results and insights were presented and disseminated. The GerOSS platform was primarily conceived as a system for the improvement of quality in the management of care.

Wagner et al [86] developed ValidAid, a context-aware system for determining the patient adherence levels to clinical recommendations when using current BP self-measurement methods and equipment. The ValidAid prototype consisted of several technical components: an adherence model, physical context sensors, health care devices, and an app. The ValidAid app implemented the adherence model; provided a user interface for the test facilitator, data processing, and communication facilities; and implemented an audio context classification.

Huberty et al [87] used Fitbit devices to describe the trajectory of PA and sedentary behavior and whether they differ among weight status in pregnant women self-identified as inactive. A total of 80 inactive pregnant women participated in this study. Different patterns were discovered, providing a first insight into the study of the influence of these parameters in pregnant women.

Jeon et al [88] evaluated 4 mobile apps that provide tailored nursing recommendations for the metabolic syndrome management (obesity, GDM, hypertension, and hyperlipidemia). Six lifestyle management and five disease-specific knowledge domains were extracted. However, the work was presented without detailed information on the design of the different apps, and the results focused only on usability measurements.

Jonas et al [89] developed a smartphone app based on the Congo Red Dot test; a simple modality to assess the presence of misfolded proteins in urine, as a diagnostic and prognostic tool for preeclampsia. Its purpose was to guide nonspecialized personnel through seven easy steps. A high agreement between manual and automated processing was calculated, but no complete clinical validation was carried out.

Marko et al [90] conducted a prospective observational study to determine the feasibility of monitoring patients remotely in prenatal care using a mobile phone app and connected digital devices. Eight women with low-risk pregnancy in the first trimester were recruited; each receiving a mobile phone app with a connected digital weight scale and BP cuff for at-home data collection for the duration of pregnancy. At-home data were assessed for abnormal values of BP or weight to generate clinical alerts to the patient and provider. As a measurement of the feasibility of the system, the engagement with the app, accuracy of remote data, efficacy of alert system, and patient satisfaction were analyzed. Automatic clinical alerts identified two episodes of abnormal weight gain with no false triggers, and patients demonstrated a high satisfaction with the system.

Torres et al [91] conducted a lifestyle intervention for Hispanic pregnant women based on two components: nutrition and PA. The nutrition component consisted of recommendations for total calories, food quantity, and improving carbohydrate and fat quality. The PA focused on limiting sedentary behavior and promoting regular movement. The intervention was delivered through individual and group sessions and phone calls. The published results focused only on identifying barriers to delivering this lifestyle intervention, and the complete clinical results were postponed for future publications.

Lange et al [92] conducted a study aimed to evaluate the readability, content, and quality of patient education materials addressing preeclampsia. Websites of US obstetrics and gynecology residency programs were searched for patient education materials. Readability, content, and quality were assessed, with a one-sample *t* test to evaluate the mean readability level compared with the recommended 6th grade reading level. As a result of this study, it was concluded that the mean readability was above the recommended 6th grade reading level. However, the content, readability, and actionability of preeclampsia patient education materials should be improved.

Fernández Arana [93] performed a review focused on the study of SMS text messages (short message service, SMS) as a health promotion tool in pregnant women. The objective of this study was to analyze the results of documented experiences in health promotion among pregnant women, through the use of SMS text messages to mobile phones in the stages of pregnancy and puerperium, presenting its future use as one more complement of telemedicine. The potential benefits of this technology, because of the characteristics of universality, mobility, instant access, and direct communication offered by these devices, was pointed out.

### Research Question 1: Which Mobile Health Solutions Have Been Used in Digital Interventions Involving Patients With Hypertensive Disorders in Pregnancy?

The different technological solutions used were grouped into websites (n=5) [69,85-87], mobile apps (n=6) [84,86-90], and others (n=1) [91]. When analyzing mobile apps, 2 were Android apps [86,88] and the other 2 iOS apps [89,90].

Only 4 studies used sensors for physiological measurements [86,87,90,91]. We found a wide range of sensors (digital weight scales, BP cuffs, context sensors, pedometers and movement sensors, or phone oximeters).

Despite the evident use of BP sensors in the study of hypertensive disorders, only 2 studies [86,90], both included in the *Diagnosis and Monitoring* category, used them.

### Research Question 2: What Are the Areas of Focus of These Mobile Health Solutions?

Two articles proposed ICT solutions that matched in two different categories [86,88]. Most of the ICT solutions proposed in selected papers were classified as *Diagnosis and Monitoring* (5/11 [85,86,88-90]).

The *Education* [69,92] and *DSS* [84,86] categories were identified in 2 of 11 included ICT solutions. Finally, the *Health Promotion* [87,88,91,93] category was proposed in 4 of the 11 included studies.

### Research Question 3: What Were the Target Groups of These Mobile Health Solutions?

Most of the analyzed ICT solutions were exclusively intended to be used by patients (7/11). These solutions were classified into the *Education, Health Promotion,* and *Diagnosis and Monitoring* categories. In three cases, solutions were intended for both patients and HCPs. All the DSSs were designed to be used by HCPs. Solutions developed exclusively for HCPs were included into the *Diagnosis and Monitoring* category.

### Research Question 4: What Types of Interventions or Studies Were Performed?

**Figure 2:**
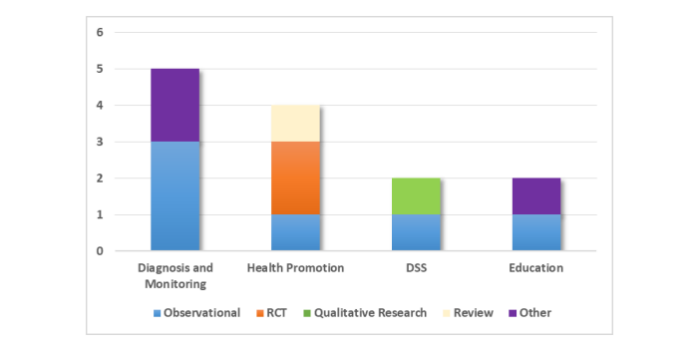
Types of interventions by categories. DSS: decision support system; RCT: randomized controlled trial.

Regarding the type of study, we found observational studies (n=4) [69,86,88,90], qualitative research (n=1) [84], RCTs (n=2) [87,91], review papers (n=1) [93], or others (n=3) [85,89,92]. Most involved a small number of participants. Figure 2 shows the distribution of the different types of interventions by categories. Observational interventions were conducted in all categories. On the other hand, RCTs were only performed in the *Health Promotion* category. Torres et al [91] performed an RCT to evaluate a lifestyle modification intervention for overweight and obese pregnant women. Huberty et al [87] conducted an RCT to analyze sedentary behavior over the course of pregnancy in inactive women.

### Research Question 5: What Were the Main Outcomes?

In general, patients using mHealth solutions felt satisfied with their prenatal care. They also felt more connected with their providers and more knowledgeable about their pregnancy. Reduced maternal anxiety was also found in relation to this enhanced knowledge. The usability of some of these solutions was assessed, resulting in medium-high scores.

On the other hand, HCPs were also satisfied with the initial prototypes reported in most cases. However, clinical evidence to include these solutions in the health care systems of many countries would require more complete RCTs, as many studies involved a small number of patients.

### Research Question 6: How Is the Research Focused on Mobile Health Solutions to Support Hypertensive Disorders in Pregnancy Distributed Across Countries?

**Figure 3:**
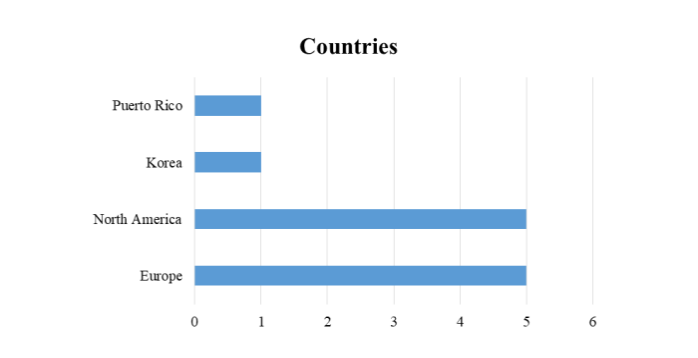
Countries of the studies analyzed.

Most of the studies reviewed were conducted in European or North American countries, as shown in Figure 3. Only 2 studies were reported in other countries (Puerto Rico and Korea).

### Research Question 7: How Is the Research Focused on Information Communication Technology Solution to Support Hypertensive Disorders in Pregnancy Distributed Over the Last 10 Years?

All articles included in this review were published recently (within the last 5 years). Most (9/11) were published in the last 2 years, indicating the current research interest in this topic.

## Discussion

### Benefits of mHealth Solutions

Pregnant women with hypertensive disorders can greatly benefit from mHealth solutions as a novel way to identify and control early symptoms, as shown in the increasing number of publications in the field. Very different benefits from the use of these mHealth solutions are found in the different studied categories described below.

### Diagnosis and Monitoring

The early detection and prevention of hypertensive complications during pregnancy are crucial. Sensors that enable measurements of relevant factors related to hypertension must be used to detect changes in women’s status and to manage them appropriately. Most of the analyzed works report the use of apps and commercial sensors for hypertension. A wide variety of commercial sensors are used. Despite the evident use of BP sensors in the study of hypertensive disorders, only 2 studies used commercial devices to monitor BP. Monitoring factors such as BP is crucial for pregnant women with hypertensive disorders to early detect and appropriately manage new symptoms and to avoid severe complications. In this sense, communication between hypertensive pregnant women and their HCPs is essential. However, only 2 papers proposed solutions, including communication features to alert physicians when a new symptom arises and manage it appropriately. This suggests that the overall process of BP monitoring can still be improved in clinical practice.

On the other hand, no use of new sensors for BP is found. Although BP is being measured from a long time, there is no precise wearable medical device that can monitor with precision BP in a continuous way. As aforementioned, simple, accurate, and low-cost new devices may be used in resource-poor environments.

Another relevant issue is that no real integration with health care systems is reported in any of the papers. Most of the studies and prototypes have been tested with a small number of participants, partly because of this lack of integration with health care systems, and therefore, larger RCTs would be necessary to find clinical evidence for these new mHealth solutions.

### Decision Support Systems and Mobile Health

DSSs are valuable tools supporting HCPs in the treatment of pregnant women with hypertensive disorders. Furthermore, DSSs could raise individual awareness and empower women in the management of their health. Although there are technologies that enable the development of DSSs, only two DSSs were found in the literature. Both were intended to be used by HCPs. No DSSs for pregnant women with hypertensive disorders have been proposed in the literature; therefore, there is a lack of evidence regarding the impact of using them on the patient’s quality of life (QoL) during pregnancy.

### Education

Patient knowledge about hypertensive disease is a relevant factor to improve health outcomes. Although there is evidence of the positive effect of educational computer-based programs in pregnant women with hypertensive disorders, we found only 2 papers focused on patient education. In addition, both educational interventions were performed using Web technologies.

Ecological momentary interventions can be conducted using mobile technologies, and educational programs could benefit from mHealth solutions. However, there is a lack of evidence of the impact of mobile educational programs on pregnant women with hypertensive diseases.

### Health Promotion

Healthy lifestyle recommendations have been included for the management of pregnant women with hypertensive disorders. In this sense, they may benefit from lifestyle interventions. Computer-based lifestyle interventions have been tested in a wide range of domains, resulting in a significant positive impact. However, only 1 paper proposing a lifestyle modification intervention for pregnant women with hypertensive disorders was found in the literature review.

On the other hand, despite the potential influence of sleep on QoL and on the risk of hypertensive disorders, no digital interventions for the management of sleep disorders have been proposed. The impact of such interventions on the QoL and their effectiveness on reducing risk of complications should be studied.

### Types of Interventions and Outcomes

Most of the included studies are focused on observational and qualitative research. The most advanced apps analyzed describe only the usability of the tool. No significant clinical study on the use of mobile apps in the control of hypertension has been reported. Only 2 RCTs are analyzed, focused on health promotion, although no medical evidence on the improvement of hypertension is reported. Therefore, there is a clear need to conduct digital interventions using the proposed solutions to clinically validate them and evaluate their outcomes. This is found to be a necessary step to support the integration of reported prototypes and protocols in health care systems and therefore improve current clinical practice.

The potential reduction of costs in the health care system would be another important outcome. However, almost all the solutions found are prototypes, and authors did not include any economic information. A complete cost analysis for each solution would be interesting, including costs related to the development, the required adaptations (translation, cultural adaptation, etc), or other required resources such as the Internet connection.

### Low- and Middle-Income Countries

Digital health is especially interesting in LMIC settings, with the delayed identification of women with hypertensive disorders because of their limited health care capability and facilities for testing and managing such patients. Using ICT-based interventions, a large number of patients could be treated at any time and place, reducing health care costs, while the quality and efficiency of care are maintained or even increased. In this sense, smartphones are becoming an interesting option because of their increasing penetration in many LMICs, their small size and weight, their communication capabilities (3G or Wi-Fi connections), their integrated sensors, and their reduced costs. Although most of the analyzed solutions were based on mobile technologies, only 2 were Android apps, which may meet the requirements for resource-poor environments of LMIC. This is very important to meet the United Nations SDG [58], particularly SDG 3: “Ensure healthy lives and promoting well-being for all at all ages, which among its targets are to reduce the maternal and neonatal mortality ratios.”

Although a mobile app can be a good tool to avoid or treat hypertension disorders in LMIC, with a limited health care budget, most of the studies have been conducted in European or North American countries. Furthermore, many of the apps (n=2) have been developed for Apple systems, which are of limited use in these countries.

### Limitations

This scoping review analyzed journal articles from five databases, but additional results may be obtained by taking into consideration conference proceedings and gray literature and by using other databases.

The results are especially interesting for LMIC, and although no more information has been found in the analyzed databases, further unpublished information and recent clinical experiences in these countries may be found.

### Conclusions

Hypertensive disorders are the most common complications during pregnancy, with gestational hypertension and preeclampsia being the leading causes of perinatal and maternal morbidity and mortality. Pregnant women with hypertensive disorders could greatly benefit from electronic health and mHealth solutions, which are useful tools for the early recognition of signs and symptoms of these hypertensive disorders and may prevent important complications.

However, there is still room for improvement in several areas such as the remote monitoring methods of BP of utmost importance in the management of gestational hypertension. Furthermore, at the moment, no real integration with health care systems is reported in the reviewed papers. Most of the reported mHealth prototypes have been tested with a small number of participants, and therefore, larger clinical trials would be necessary for these new mHealth solutions to be supported for integration in health care systems.

In LMIC, mHealth solutions may overcome access-limiting factors and the lack of skilled medical professionals and low health care budget. Using ICT-based interventions, a large number of patients could be treated at any time and place, reducing health care costs, while the quality and efficiency of care are maintained or even increased. Therefore, there is a clear need for simple mHealth solutions specifically developed for resource-poor environments that meet the United Nations SDG [58], particularly SDG 3: “Ensure healthy lives and promoting well-being for all at all ages, which among its targets are to reduce the maternal and neonatal mortality ratios.” A complete clinical trial on the use of mHealth solutions for the diagnosis and monitoring, medical treatment, or prevention of hypertensive disorders and their effects in pregnant women would also be necessary in these countries to improve current clinical practice and demonstrate such improvement.

## Appendix

Multimedia Appendix 1 Summary of data collected.

## Abbreviations

BP: blood pressure
CVD: cardiovascular disease
DSS: decision support system
HCP: health care professional
ICT: information and communication technology
LMIC: low- and middle-income countries
mHealth: mobile health
PA: physical activity
RCT: randomized controlled trial
SDG: Sustainable Development Goal
SMS: short message service
QoL: quality of life
