# Supplementary material for: Mobile Health Solutions for Hypertensive Disorders in Pregnancy: Scoping Literature Review
Source: JMIR Mhealth Uhealth. 2018 May 30;6(5):e130. doi: 10.2196/mhealth.9671 (PMC6000483; doi:10.2196/mhealth.9671)
Supplement: Multimedia Appendix 1 [file mhealth_v6i5e130_app1.pdf]

Table 1: Summary of articles: publication data

Article	Journal abbrev.	Pub. Year	Ranking	Category *
[69]	J. Obstetric & Gyn. Can.	2013		Ed
[84]	Int. J. Med. Inform.	2015	Q1	DSS
[85]	Methods Inf. Med.	2015	Q1	D&M
[86]	Methods Inf. Med.	2014	Q1	D&M DSS
[87]	Prev. Med. Rep.	2016		HP
[88]	Stud. Health Tec. Inf.	2016		D&M HP
[89]	J. Am. Med. Inform. Assn.	2015	Q1	D&M
[90]	JMIR Res. Protoc.	2016		D&M
[91]	Contemp. Clin. Trials Comm.	2016		HP
[92]	Hypertens. Pregnancy	2015	Q4	Ed
[93]	Metas Enf	2016		HP

* Category code: Ed: Education; DSS: Decision Support System; D&M: Diagnosis and Monitoring; HP: Health Promotion.

Table 2: Information regarding interventions

Article	Type of intervention	Country	Context	Num. Participants
[69]	Prospective Study	Canada	Home<br>Clinical	18 pregnant women
[84]	Qualitative Research	Italy<br>Spain	Unknown	29 patients (10 Atrial fibrillation patients + 19 GDM women)<br>Care providers
[85]	Database Analysis	Germany	Clinical	910 cases
[86]	Observational	Denmark	Clinical	41 patients
[87]	RCT	USA	Daily	80 inactive pregnant women
[88]	Usability Study	Korea	Unknown	Unknown
[89]	Multistage tests	Germany	Unknown	218 first stage<br>273 second stage
[90]	Prospective Observational trial	USA	Home	6 pregnant women
[91]	RCT	Puerto Rico	Home<br>Clinical	200
[92]	Website Evaluations	USA	N/A	114 websites
[93]	Review	N/A	N/A	N/A

Table 3: Type of ICT solution

Article	Software	Platform	Sensors	Users
[69]	Web	N/A	None	Patients

[84]	Mobile	Unknown	None	Patients<br>Physicians
[85]	Web	N/A	None	Physicians
[86]	Mobile<br>(Tablet app)<br><br>Web	Android	Blood pressure<br>Context sensors	Patients<br>Physicians
[87]	Mobile<br>Web	Unknown	Movement sensors	Patients
[88]	Mobile	Android	None	Patients
[89]	Mobile	iOS	None	Patients<br>Physicians
[90]	Mobile	iOS	Digital weight scale<br><br>Blood pressure	Patients
[91]	Other	Unknown	Pedometer	Patients
[92]	Web	N/A	None	Patients
[93]	Mobile (N/A)	N/A	N/A	Patients

Table 4: Features of ICT solution

**Article**	**Features**	**Communication**	**Content Confidence**
[69]	Education (placental knowledge)	N/A	Experts
[84]	Clinical guideline-based recommendations<br>Decision support system<br>Semantic data integration into PHR	Notifications (patients)<br><br>Alerts (physicians)	Clinical guidelines

	Intelligent data analysis		
[85]	Documentation	N/A	Scientific committee
[86]	Adherence model<br>User interface<br>Data processing<br>Communication<br>Audio context classification	N/A	N/A
[87]	Monitoring of physical activity	Notifications	N/A
[88]	Management of metabolic syndrome	Unknown	Unknown
[89]	Diagnosis through image processing	Mail	N/A
[90]	Educational information	Notifications (patients)<br>Mail (physicians)	3 board-certified obstetrician-gynecologists
[91]	Education	N/A	Experts
[92]	Patient educational materials	N/A	N/A
[93]	Use of SMS	N/A	N/A
